# The effects of Rashba spin-orbit and non-linear electron-phonon interactions on impurity-induced bound states in BCS superconductors

**DOI:** 10.1038/s41598-026-51558-z

**Published:** 2026-05-28

**Authors:** B. Navashreya, Narasimha Raju Chebrolu, I. V. Sankar, Aalu Boda

**Affiliations:** 1 Department of Physics, Central University of Karnataka, Kalaburagi, Karnataka 585367 India; 2 Department of H & S, CVR College of Engineering, Rangareddy, Telangana 501510 India; 3 Department of Physics, National Institute of Technology Warangal, Hanamkonda, Telangana 506004 India

**Keywords:** Materials science, Physics

## Abstract

We have theoretically studied the influence of linear, non-linear electron-phonon interactions and Rashba spin orbit interaction on formation of the localized magnetic moment and subgap Yu-Shiba-Rusinov bound state for a single-level quantum impurity embedded in an s-wave Bardeen-Cooper-Schrieffer superconductor, modeled by single-impurity Anderson-Holstein Hamiltonian. We have used time-scale separation approach combined with Bogoliubov transformation and modified Lang-Firsov transformation to decouple quadratic and linear electron-phonon interactions present on impurity. Then we used the Kikuchi-Morita cluster variation method for the calculation of ground state energy of the system. We theoretically studied the influence of Rashba spin-orbit interaction on the correlation effects of magnetic impurity embedded in an s-wave superconductor. Further, we analyze transition from single to bipolaron phase transition at the impurity site as a function external tunable Rashba spin orbit interaction strength.

## Introduction

Impurities invariably always exist in condensed matter systems, such as superconductors and semiconductors. Impurities are frequently thought of as a bother that ruins a clean system’s qualities and makes it more difficult to comprehend them. The magnetic impurity induced bound states within the superconducting energy gap, so-called Yu-Shiba-Rusinov (YSR) bound states, inside the energy gap of a conventional Bardeen-Cooper-Schrieffer (BCS) superconductor (SC) by breaking Cooper pairs locally in the vicinity of impurity, as a result, that affects the optical and electromagnetic properties of the SC^1–8^. On the other hand, impurities can serve as special atomic-scale probes of the host system’s ground state and are frequently necessary to achieve desired physical effects^9–16^. These intra-gap bound states have recently been suggested to give rise to emergent Majorana zero-energy modes at the end-points of chains of magnetic impurities in systems with spin-orbit coupling^17–19^. As a result, impurity-induced bound states in SCs have attracted a lot of attention in both theory and experiment^20–22^.

Recent advancements in nano-fabrication techniques made it possible to construct the hybrid devices consisting of a quantum dot (QD) or molecule (which can be treated as a quantum impurity) with discrete energy levels coupled to metallic or superconducting leads. In literature people used the scanning tunnelling microscopy and scanning tunneling spectroscopy techniques to identify the impurity induced bound states in systems like Gd, and Mn atoms on the surface of superconducting Nb, magnetic molecules, magnetic nanostructures, magnetic islands deposited on SCs, and magnetic molecular junctions with proximity-induced superconductivity. The SC-QD-SC hybrid device’s electrical and transport features are being experimentally studied by several groups^23–25^. In a carbon-nanotube QD-SC system, Maurand et al.^26^ investigated the competition between proximity-induced pairing and strong electron-electron (*el-el*) interaction. It was also suggested by the aforementioned research that an impurity band with non-trivial topological character is formed by the bound states induced by the magnetic impurities. These bound states enable the construct of the noise-resilient qubits, possible building blocks for topological quantum computers. It also demonstrates that counting the number of induced subgap states is a simple way to explore the symmetry of the order parameter^27^. Furthermore, the physical characteristics of an impurity in SC provide important insights into the bulk and can therefore be a critical probe for determining the bulk superconducting state’s features. Despite the significant advancements in theory and experimentation, the impact of local electron-phonon (*el-ph*)(Holstein type) and spin-orbit interactions on impurity bound states, the YSR states in SCs, remains poorly understood.

In this work we have considered magnetic QD with Rashba spin orbit interaction (RSOI), linear and non-linear *el-ph* interactions and *el-el* interaction embedded in a BCS superconductor to analyze the effects of these interactions on impurity bound state and YSR state. The paper is organized as follows: In section "Model Hamiltonian and theoretical formulation" we introduce the AH model to describe the system. In section "Ground state energy" we derive the ground state energy of the impurity bound state using the cluster variation method. In sections "Large gap limit" and "Results and discussions" we discuss the effective Hamiltonian in the subgap limit and the numerical results, respectively, and finally end the paper with the summary and conclusions in section "Summary and conclusion".

## Model Hamiltonian and theoretical formulation

**Fig. 1:**
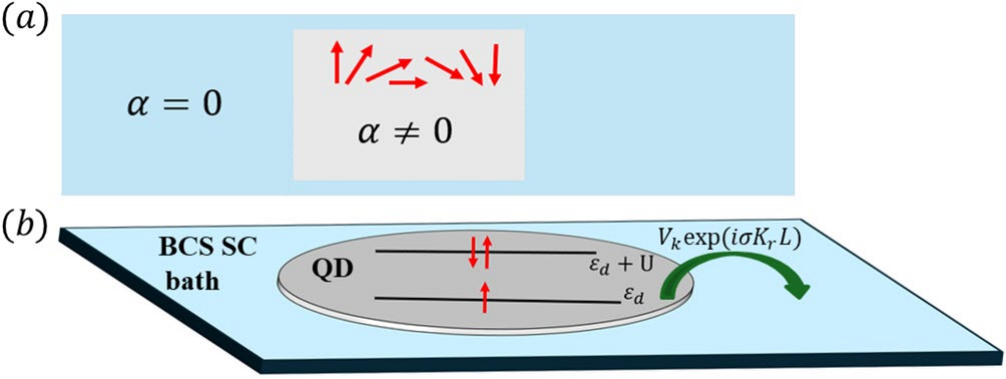
The system consists of QD embedded on a BCS SC bath as shown in the figure. (**a**) Rashba SOI restricted to QD as shown. (**b**) *s-d* interaction between QD and BCS SC as shown.

Based on the description of the system, the total Hamiltonian using the Anderson-Holstein model can be given as

1 $$\begin{aligned} H = H_{SC} + H_{QD} + H_{t}\,, \end{aligned}$$

where,

2 $$\begin{aligned} H_{SC}=\sum _{k\sigma } \varepsilon _{k\sigma }\,n_{k\sigma } + \Delta _{s} \sum _{k\sigma }(c_{k\sigma }^{\dagger }c_{-k\bar{\sigma }}^{\dagger } + h.c.) \end{aligned}$$

represents the Hamiltonian of the SC where first term is kinetic energy with the number operator $n_{\sigma }=c_{k\sigma }^{\dagger }c_{k\sigma }$ with momentum *k* and spin index $\sigma$, second term represents pairing Hamiltonian within the mean field approximation, where $\Delta _{s}$ being the superconducting gap parameter. The second term,

3 $$\begin{aligned} H_{QD}&= \sum _{\sigma }\left[ \varepsilon _{d} + g_1\,\omega _{o}(b^{\dagger }+b)+g_2\,\omega _{o}(b^{\dagger }+b)^{2}\right] n_{d\sigma }\nonumber \\&\quad + U\,n_{d\uparrow }n_{d\downarrow } + \omega _{o}b^{\dagger }b\, , \end{aligned}$$

is Hamiltonian of the quantum dot with single energy level $\varepsilon _{d}$, the number operator $n_{d\sigma } = c_{d\sigma }^\dagger \,c_{d\sigma }$, and local Coulomb interaction strength *U*. We have considered the local *el-ph* interaction upto quadratic order in the displacement $(b^{\dagger }+b)$, with phonon creation (annhilation) operator $b^{\dagger } (b)$, with frequency $\omega _{o}$. Here $g_1$, and $g_2$ represents the linear and quadratic *el-ph* interaction strengths and let $\hbar = 1$. As shown in Fig. 1, we have considered the RSOI present only at the QD. The third term in the total Hamiltonian is given by^28^,

4 $$\begin{aligned} H_{t} = \sum _{k\sigma } \left( V_{k}\,c_{d\sigma }^{\dagger }c_{k\sigma }\,e^{i\sigma K_r L} + h.c\right) . \end{aligned}$$

represents the hybridization (*s-d* interaction) between QD and superconducting bath, where $V_k$ is hybridization interaction strength. The spin-dependent, non-commutative phase factor is due to RSOI with $K_r= \alpha \,m^{*}\hbar ^{-2}$, $\alpha$ being RSOI coefficient, *L* being diameter of the QD, the spin index $\sigma =\uparrow \,\downarrow$. The RSOI is assumed to exist only within the QD region. It leads to the mixing of spin-up and spin-down states when electron hopping from conduction band to the QD. The experimentally observed value of RSOI strength $\alpha \sim 3\times 10^{-11}\,eV\,m$ for some semiconductors^29–31^. Then, $K_{r}=m^{*}\alpha \hbar ^{-2}\approx 0.015\,nm^{-1}$ for $m^{*}=0.036\,m_{e}$. If you consider the QD size around $10-100\, nm$, then $K_{r}L=0.15-1.5$. Therefore, we vary the $K_{r}L$ value from 0 (absence of RSOI) to $\pi$ in our calculation. The *el-el* interaction strength *U* (required charging energy for double occupation) in a typical QD can be calculated as^32^ $U\simeq e{^2}/\epsilon (0)L$, with $\epsilon (0)$ is static dielectric constant. For GaAs QD with diameter $20\, A^{o}$, the optical phonon energy^33^ $\omega _{0}=35\,$meV and $\epsilon (0)\simeq <span class='crossLinkCiteEqu'>13</span>$, produces $U\simeq <span class='crossLinkCiteEqu'>2</span>\omega _{0}$.

Recent research has revealed that time-scale separation in molecular junctions causes non-linear transport behaviour in molecular devices^34,35^. Thus, the impurity electron number and the phonon operators can be expressed as the sum of their fluctuation and steady-state expectation value as follows:

5 $$\begin{aligned} n_{d\sigma }=\bar{n}_{d\sigma } + \delta \,n_{d\sigma }\,,\qquad b = \bar{b} + \delta \,b. \end{aligned}$$

Substituting Eq.(5) in the *el-ph* interaction term of QD term Hamiltonian, we get

6 $$\begin{aligned}&\left[ g_1\omega _{o}(b^{\dagger }+b)+g_2\omega _{o}(b^{\dagger }+b)^2\right] \sum _{\sigma }\bar{n}_{d\sigma }\nonumber \\&\quad +\left[ g_1\,\omega _0\sqrt{2\omega _{o}}\,\bar{x}_{b} +2g_2\omega _{o}^{2}\,\bar{x}_{b}^{2}\right] \sum _{\sigma }n_{d\sigma }\, , \end{aligned}$$

where, $\bar{x}_{b} =\left( \bar{b}^{\dagger }+\bar{b}\right) /\sqrt{2\omega _{o}}$. To obtain the Eq.(6), we add a constant term $\left( g_1\,\omega _{o}\sqrt{2\omega _{o}}\,\bar{x}_{b} +2g_2\omega _{o}^{2}\,\bar{x}_{b}^{2}\right) \sum _{\sigma }\bar{n}_{d\sigma }$, independent of time and it does not effect the dynamics of the system and the terms containing $\delta x_{b}\delta n_{d\sigma }$ and $\delta x_{b}^2\,\delta n_{d\sigma }$ is neglected as their contribution is much small. to proceed further we apply Bogoliubov transformation: $a = b\, \cosh \theta + b^{\dagger }\,\sinh \theta$, with the transformation parameter $\theta$ given by $e^{\theta }=\sqrt{\omega _{a}/{\omega _{o}}}$ and the frequency of the dressed vibrational mode is given by,

7 $$\begin{aligned} \omega _{a}=\omega _{o}\,\sqrt{1+4g_2\sum _{\sigma }\bar{n}_{d\sigma }}. \end{aligned}$$

After rearranging all the terms, the renormalized Hamiltonian is given by,

8 $$\begin{aligned}&H_{re} = \sum _{k\sigma } \varepsilon _{k\sigma }\,n_{k\sigma } + \Delta _s\sum _{k\sigma }(c_{k\sigma }^\dagger c_{-k\bar{\sigma }}^\dagger + h.c) \nonumber \\&\quad + \sum _{\sigma }\left[ \tilde{\varepsilon }_{d} + \tilde{g_1}\,\omega _{a}(a^{\dagger }+a)\right] n_{d\sigma }+ U\,n_{d\uparrow }n_{d\downarrow } + \omega _{a}a^{\dagger }a\nonumber \\&\quad + \sum _{k\sigma } \left( V_{k}\,c_{d\sigma }^{\dagger }c_{k\sigma }\,e^{i\sigma K_r L} + h.c\right) , \end{aligned}$$

where, $\tilde{g}_{1} = g_1\,e^{-3\theta }$, and the renormalized QD energy level due the linear and quadratic *el-ph* interactions given by, $\tilde{\varepsilon }_{d}=\varepsilon _{d} + \tilde{g}_1\omega _{a}\sqrt{2\omega _{a}}\,\bar{x}_{a} + 2g_2\omega _{a}^{2}\bar{x}_{a}^{2}$ with $\bar{x}_{a}$ the steady state average phonon displacement. From the expression $\tilde{g}_{1}$ and $\omega _{a}$, we can observe that a small positive (negative) nonlinear *el-ph* interaction strength reduces (increases) the effective linear *el-ph* interaction strength and hardens (softens) the effective phonon frequency. With the help of Heisenberg-Langevin equation, one can write,

9 $$\begin{aligned} \frac{d^2 x_a(t)}{dt^2} = - \omega _{a}^2\,x_a(t) - \tilde{g}_{1}\omega _{a}\sqrt{2\omega _{a}}\,\sum _\sigma \bar{n}_{d\sigma }, \end{aligned}$$

giving the solution as,

10 $$\begin{aligned} x_a(t)&= x_a(t_0)\cos {(\omega _{0}t)} + \frac{p_a(t_0)}{\omega _{a}}\,\sin {(\omega _{a}t)} \nonumber \\&+ \int d\tau \, D_{0}^{R}\left( t-t'\right) \tilde{g}_{1}\omega _{a}\sqrt{2\omega _{a}}\,\sum _\sigma \bar{n}_{d\sigma }, \end{aligned}$$

where $D_{0}^{R}$ is the non-interacting retarded phonon Green’s function which satisfies the equation:

11 $$\begin{aligned} \left( \frac{d^2}{dt^2} + \omega _{a}^2 \right) D_{0}^{R}\left( t-t'\right) = -\delta \left( t-t' \right) . \end{aligned}$$

Using, $D_{0}^{R}\left( \varepsilon \right) = 1/\left( \varepsilon ^2 - \omega _{a}^2 \right)$ and $\omega _{a}$ value given in Eq. (7), the steady state average displacement is obtained as,

12 $$\begin{aligned} \bar{x}_{a}&= \tilde{g}_{1}\omega _{a}\sqrt{2\omega _{a}}\,\sum _\sigma \bar{n}_{d\sigma }\,D_{0}^{R}\left( \varepsilon = 0\right) \nonumber \\&= -\frac{\tilde{g}_{1}\sqrt{2\omega _{a}}\,\sum _\sigma \bar{n}_{d\sigma }}{\left( 1+4g_2\sum _{\sigma }\bar{n}_{d\sigma }\right) \omega _{0}}, \end{aligned}$$

hence, we obtain renormalized QD energy level. We use the generalized Lang-Firsov transformation^36,37^ with a variational parameter $\left( 0\le \zeta \le 1 \right)$, to decouple the linear *el-ph* interaction. $\zeta$ provides a physical measurement of the polarization potential that the *el-ph* interaction creates. It will be ascertained by minimizing the ground state energy. The generator $S = \zeta \,\tilde{g}_{1}(a^{\dagger }-a)\sum _{\sigma } n_{d\sigma }$ is used to obtain the transformed Hamiltonian $\bar{H} = e^{S}H_{re}e^{-S}$,

13 $$\begin{aligned}&\bar{H} = \sum _{k\sigma } \varepsilon _{k\sigma }\,n_{k\sigma } + \Delta _s\sum _{k\sigma }(c_{k\sigma }^\dagger \,c_{-k\bar{\sigma }}^\dagger + h.c)\nonumber \\&\quad + \sum _{\sigma }\left[ \tilde{\tilde{\varepsilon }}_{d} + \tilde{g}_{1}\,\omega _{a}(a^{\dagger }+a)\right] n_{d\sigma }+ \tilde{U}\,n_{d\uparrow }n_{d\downarrow } + \omega _{a}a^{\dagger }a \nonumber \\&\quad + \sum _{k\sigma } \left( \tilde{V}_{k}\,c_{d\sigma }^{\dagger }c_{k\sigma }\,e^{i\sigma K_r L} + h.c\right) , \end{aligned}$$

and,

14 $$\begin{aligned} \tilde{\tilde{\varepsilon }}_{d}&= \tilde{\varepsilon }_{d} - \varepsilon _{p} = \tilde{\varepsilon }_{d} - \tilde{g}_1^{2}\omega _{a}\,\zeta (2-\zeta )\nonumber \\ \tilde{U}&= U - 2\tilde{g}_1^{2}\omega _{a}\zeta \,(2-\zeta ) \nonumber \\ \tilde{V}_{k}&= V_{k}\, e^{\tilde{g}_{1}(a-a^{\dagger })\zeta }. \end{aligned}$$

The effects of *el-ph* interaction i.e the reduction in the QD energy level by a polaronic energy $\varepsilon _{p}$, the phonon mediated electron tunneling strength $\tilde{V}_{k}$, and the renormalized Coulomb interaction strength are easily noticeable. After the zero phonon averaging to the transformed Hamiltonian we get,

15 $$\begin{aligned}&H_{eff} = \sum _{k\sigma } \varepsilon _{k\sigma }\,n_{k\sigma } + \Delta _s\sum _{k\sigma }(c_{k\sigma }^\dagger \,c_{-k\bar{\sigma }}^\dagger + h.c)\nonumber \\&\quad + \sum _{\sigma }\tilde{\tilde{\varepsilon }}_{d} n_{d\sigma } + \tilde{U}\,n_{d\uparrow }n_{d\downarrow } \nonumber \\&\quad + \sum _{k\sigma } \left( \tilde{V}_{k}\,c_{d\sigma }^{\dagger }c_{k\sigma }\,e^{i\sigma K_r L} + h.c\right) . \end{aligned}$$

We use localized polaron approximation^38,39^, valid only when $g_1\gg V_{k}$, as a result $\tilde{V}_{k} = V_{k}\,e^{-\tilde{g}_{1}\zeta (N_{ph}+1/2)}$ in Eq. (12) to obtain the effective Hamiltonian. Here $N_{ph}=\left[ \exp (\beta \omega _{a})-1 \right] ^{-1}$ represents the phonon population on the molecule with $\beta =1/k_{B}T$. Now the effective Hamiltonian can be separated into the electronic and phononic parts.

## Ground state energy

The ground state energy is obtained by evaluating the free energy at zero temperature. The variational free energy of a system is constructed by introducing correlations among different particles (continuum and localized impurity electrons), which are then expressed in terms of the Hamiltonian and the trial reduced density matrix as, $F=Tr[\rho _t(H_{eff}\,+ K_BT\,ln\,\rho _t)]$ where *Tr* denotes the trace operation, $\rho _t$ denotes normalized density matrix, $K_B$ denotes the Boltzmann constatnt and *T* being absolute temperature. By expressing the trial free energy in such a way, we can introduce approximations in a systematic manner. The number of terms that we retain depends on the nature of the problem. If the trial free energy is truncated after keeping correlations among “*n*” different particles of the system, we say we are treating clusters of “*n*” particles. In our work we have considered up to two particle cumulants, so we have considered cluster of two particles $n_k$ and $n_d$. The free energy can be written as^40–42^,

16 $$\begin{aligned}&F= \sum _j tr_j \,h^{(1)}(j)\rho _{t}^{(1)}(j) + \sum _{j>k} tr_{j,k}\, h^{(2)}(j,k)\rho _{(t)}^{(2)}(j,k) \nonumber \\&+ K_BT \left( \,\sum _{j} \gamma ^{(1)}(j) + \sum _{j>k}\gamma ^{(2)}(j,k)\right) \end{aligned}$$

where $h^{(1)}(j)$ and $h^{(2)}(j,k)$ represent single and two particle terms in effective Hamiltonian respectively, $\rho ^{(1)}(j)$, $\rho ^{(2)}(j,k)$ and $\rho ^{(2)}(k,-k)$ represent one and two particle reduced density matrices. $\gamma ^{(1)}(j)$ and $\gamma ^{(2)}(j,k)$ are one and two particle cumulants given by:

17 $$\begin{aligned}&\gamma ^{(1)}(j) = tr_j\, \rho _t^{(1)}(j) \, ln \rho _t^{(1)}(j), \nonumber \\&\gamma ^{(2)}(j,k) = tr_j\, \rho _t^{(2)}(j,k)\, ln \rho _t^{(2)}(j,k) - \gamma ^{(1)}(j) - \gamma ^{(1)}(k) , \nonumber \\&\gamma ^{(2)}(k,-k)= tr_j \rho _t^{(2)}(k,-k) ln \rho _t^{(2)}(k,-k)\nonumber \\&\,\,\, - \gamma ^{(1)}(k) - \gamma ^{(1)}(-k) \end{aligned}$$

Here we consider upto second order cumulants, then free energy at $T=0 K$ is ground state energy of the system given as:

18 $$\begin{aligned}&E_0 = \sum _{k\sigma }\varepsilon _k\langle n_{k\sigma }\rangle + \sum _{\sigma } \tilde{\tilde{\varepsilon }}_{d\sigma }\langle n_{d\sigma }\rangle + \tilde{U}\langle n_{d\sigma }\rangle \langle n_{d\bar{\sigma }}\rangle \nonumber \\&\quad - 2|\tilde{V}_{k}|\sum _{k\sigma } \left[ \langle n_{k\sigma } \rangle \langle n_{d\sigma } \rangle \left( 1- \langle n_{k\sigma }\rangle \right) \left( 1-\langle n_{d\sigma }\rangle \right) \right] ^{1/2} e^{i\sigma K_r L}\nonumber \\&\quad -2\Delta _s\sum _{k\sigma } \left[ \langle n_{k\sigma } \rangle \langle n_{-k\bar{\sigma }}\rangle \left( 1- \langle n_{k\sigma }\rangle \right) \left( 1- \langle n_{-k\bar{\sigma }} \rangle \right) \right] ^{1/2} \end{aligned}$$

We have used mean field level decoupling schemes as following: $\left\langle n_{d\sigma }n_{d-\sigma } \right\rangle = \left\langle n_{d\sigma } \right\rangle \left\langle n_{d-\sigma } \right\rangle , \left\langle n_{k\sigma }n_{d\sigma } \right\rangle =\left\langle n_{k\sigma } \right\rangle \left\langle n_{d\sigma } \right\rangle , \left\langle n_{k\sigma } n_{-k\bar{\sigma }}\right\rangle = \left\langle n_{k\sigma }\right\rangle \left\langle n_{-k\bar{\sigma }} \right\rangle$. We considered that the correlations between *d* electrons of opposite spin, and between conduction and *d* electrons are small. But we considered the correlation terms like $\langle c_{k\sigma }^{\dagger }c_{d\sigma }\rangle$ while calculating the single and two particle entropy terms. We shall minimize $E_0$ and compute $\left\langle n_{d\sigma } \right\rangle$, $\left\langle n_{k\sigma }\right\rangle$ and magnetic moment $m=\left( \left\langle n_{d\uparrow } \right\rangle -\left\langle n_{d\downarrow } \right\rangle \right)$ using the following:

19 $$\begin{aligned}&\eta _{d\sigma }=\left\langle n_{d\sigma }\right\rangle \left( 1-\left\langle n_{d\sigma }\right\rangle \right) = \left( 1- m^2 \right) /4 \nonumber , \\&\eta _{k\sigma } = \left\langle n_{k\sigma } \right\rangle \left( 1 - \left\langle n_{k\sigma }\right\rangle \right) \nonumber , \\&\varepsilon _{0\sigma }= 2|\tilde{V}_{k}|\sum _{k\sigma }\sqrt{\eta _{k\sigma }}\,e^{i\sigma K_r L}, \nonumber \\&\tilde{\varepsilon }_{k} = \left( \varepsilon _k^2 - \Delta _s^2 \right) ^{1/2}. \end{aligned}$$

After calculations, we obtain the following:

20 $$\begin{aligned} \left\langle n_{k\sigma }\right\rangle = \frac{1}{2}\left[ 1-\tilde{\varepsilon }_k\left( \tilde{\varepsilon }_k + 4|\tilde{V}_{k}|^2\sum _{\sigma }\eta _{d\sigma }cos^2(K_rL)\right) ^{-1/2} \right] \end{aligned}$$

21 $$\begin{aligned} E_0&= \frac{\omega _D}{2}\left( \omega _D^2-\Delta _s^2 \right) ^{1/2}+ \tilde{\tilde{\varepsilon }}_{d\sigma }\nonumber \\&\qquad +\frac{\Delta _s^2}{2}\,\,ln \left( \frac{\omega _D + \left( \omega _D^2 -\Delta _s^2 \right) ^{1/2}}{\Delta _s} \right) + \tilde{U}\left( \frac{1-m^2}{4} \right) \nonumber \\&\qquad -\frac{1}{2}\left[ \right. \omega _{D}\,\sqrt{\omega _{D}^{2}+Z}-2\Delta _{s}|\tilde{V}_{k}|cos(K_{r}L)\left( \frac{\sqrt{1-m^2}}{2}\right) \left. \right] \nonumber \\&\qquad +\frac{1}{2}\left[ |\tilde{V}_{k}|^{2}cos^{2}(K_{r}L)(1-m^2)-\Delta _s^2\right] \times \nonumber \\&\quad \left[ \right. sinh^{-1}\left( \frac{\omega _D}{\sqrt{Z}} \right) -sinh^{-1}\left( \frac{\Delta _s}{\sqrt{Z}}\right) \left. \right] \end{aligned}$$

where, $Z=|\tilde{V}_{k}|^{2}cos^{2}(K_{r}L)(1-m^2)-\Delta _{s}^2$. We have considered the half filled case in the QD where $N=\left\langle n_{d\uparrow }\right\rangle + \left\langle n_{d\downarrow }\right\rangle = 1$, which corresponds to symmetric case $\varepsilon _{d\sigma } = -U /2$. Where $n_{k\sigma }$ is the conduction electron distribution and the value of $\varepsilon _{0\sigma }$ in Eq.(18) has been obtained by replacing the summation over *k* by an integral over $\varepsilon _{k}$ using the SC density of states $\rho (\varepsilon _{k})/\rho (0) =\left( \varepsilon _{k}/\tilde{\varepsilon }_{k}\right) \theta (|\varepsilon _{k}|-\Delta )$ with $\omega _D$(Debye frequency) as the cutoff energy of the conduction band.

The energy difference between a BCS SC with a magnetic impurity and the unaltered system at zero temperature is defined as the binding energy given by,

22 $$\begin{aligned} W&= \varepsilon _d - E_0 \nonumber \\&\quad = \tilde{\tilde{\varepsilon }}_{d\sigma } - E_0 \nonumber \\&\qquad - \left( \tilde{g}_{1}\omega _{a}\sqrt{2\omega _{a}}\,\bar{x}_{a} + 2g_2\omega _{a}^{2}\bar{x}_{a}^{2} -\tilde{g}_{1}^{2}\omega _{a}\,\zeta (2-\zeta )\right) \end{aligned}$$

The ground state energy of this system is lower when compared to unperturbed system.

## Large gap limit

The superconducting atomic limit represents the limit $D \rightarrow \infty$, $\Delta _s \rightarrow \infty$ and this may be regarded as the low frequency expansion i.e $\Delta _s \gg \omega _D$. In this limit, the system’s effective Hamiltonian^43^ is

23 $$\begin{aligned} H_{eff} = \sum _{\sigma } {\tilde{\xi }}_d n_{d\sigma } - {\tilde{\Gamma }} \left( d_{\uparrow }^\dagger d_{\downarrow }^\dagger + h.c \right) + \frac{\tilde{U}}{2}\left( \sum _{\sigma } n_{d\sigma } - 1 \right) ^{2}, \end{aligned}$$

where,

24 $$\begin{aligned} \tilde{\xi }_{d} = \left( \tilde{\tilde{\varepsilon }}_{d} + \frac{\tilde{U}}{2}\right) ; \quad \tilde{\Gamma } = 2\pi |\tilde{V}_{k}|^{2} cos^{2}(K_rL) \rho (0). \end{aligned}$$

$\tilde{\xi }_{d}$ is the renormalized shifted energy level of the dot giving account for the particle hole symmetry for $\tilde{\xi }_{d} = 0$ and $\tilde{\Gamma }$ can be viewed as the renormalized coupling strength between the dot and BCS SC bath with $\rho (0)$ being the constant density of states. Without the interaction term in Eq. (22), $H_{eff}$ can be diagonalized using Bogoliubov transformation, which shows an exact correspondence to the BCS Hamiltonian. Here the Coloumb interaction results in an extra energy shift of $\frac{\tilde{U}}{2}$ for the empty $(|0\rangle )$ and bipoloronic $(|D\rangle )$ state of the QD. The Bogoliubov quasi-particle operators are defined as,

25 $$\begin{aligned} \gamma = u d_{\uparrow } - v^* d_{\downarrow }^\dagger \nonumber \\ \gamma ^\dagger = u d_{\uparrow }^\dagger - v^* d_{\downarrow } \end{aligned}$$

With this transformation, we readily obtain the excitation energies $E_d =\pm \sqrt{\tilde{\xi }_{d}^2 + \tilde{\Gamma }^2}$. The coupling strength $\tilde{\Gamma }$ mixes the empty state and bipoloron state, resulting in the new superposed eigenstates given by

26 $$\begin{aligned} & |\psi _+\rangle = u |D\rangle + v^{*} |0\rangle \quad \nonumber \\ & |\psi _-\rangle = - v^{*}|D\rangle + u |0\rangle , \end{aligned}$$

where *u* and $v^{*}$ given by,

$$\begin{aligned} u^2 = \frac{1}{2}\left( 1 + \frac{\tilde{\xi }_{d}}{E_d}\right) ,\quad \quad {v^{*}}^2 = \frac{1}{2}\left( 1 - \frac{\tilde{\xi }_{d}}{E_d}\right) . \end{aligned}$$

The four eigen states of $H_{eff}$ are the poloron states, $|\uparrow \rangle$ and $|\downarrow \rangle$ with energy $\tilde{\xi }_{d}$ and the BCS like states given in Eq. (26), with energies $E_{\pm } = (\pm E_d \, + \frac{\tilde{U}}{2})$^44^. The QD’s state in the large-gap limit is thus the consequence of a competition between the renormalized Coulomb interaction and the local pairing that is characterized by the hybridization strength $(\tilde{\Gamma })$ and the proximity effect.

## Results and discussions

**Table 1:** Summary of parameters.

$g_2$	Quadratic *el-ph* strength	0 to $\pm 0.2$^45–48^
$V_k$	Tunneling strength	1.5^49^
$\Delta _s$	Superconducting gap parameter	1
$g_1$	Linear *el-ph* strength	0 to 1.5^50^
$\alpha = \dfrac{K_{r}\hbar ^{2}}{m^{*}}$	RSOI parameter	0 to $\pi$^29–31^

In our calculations, we have measured all energy quantities in units of Debye energy $\hbar \,\omega _D$ and set it equal to 1. The range of Debye energy is in meV. Unless stated otherwise, we have considered $U=4$ and the other parameter values given in table 1. It has been determined that the quadratic *el-ph* coupling strength $g_{2}$ is both positive and negative, and its values fall between 0 and 0.2. One of the primary goals of this work is to comprehend how the RSOI along with the quadratic *el-ph* interaction affects the binding energy of the bound state and the local magnetic moment on the magnetic impurity in a BCS superconductor.

**Fig. 2:**
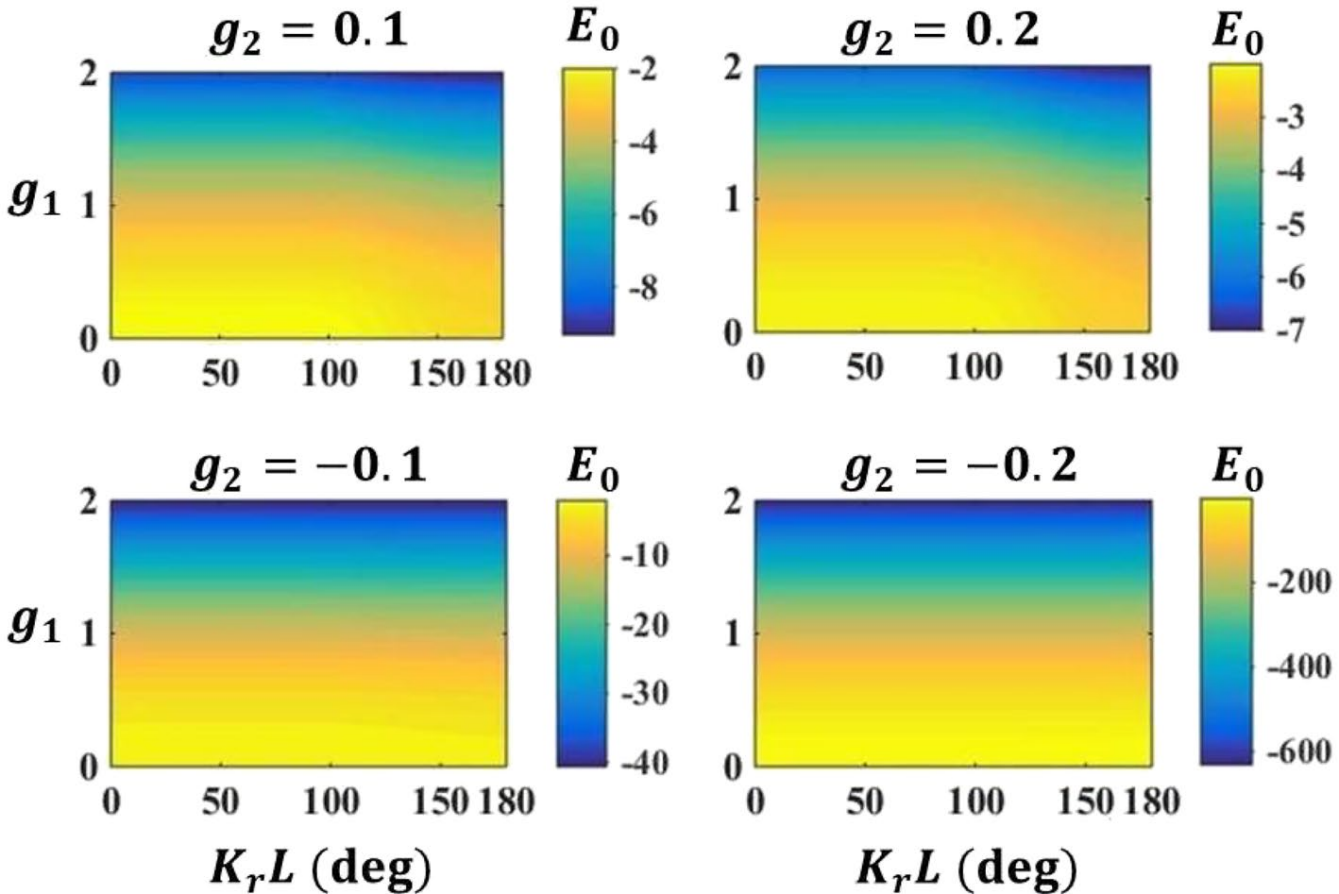
Variation of $E_0$ as a function of $g_1$, and $K_{r}L$ for different values of $g_{2}$. Ground state energy $E_0$ is observed to decrease with increasing $g_1$ and decreasing $g_2$.

We showed the $E_{0}$ as a function of $g_{1}$ and $K_{r}L$ for various values of $g_{2}$ in Fig. 2 to see the cumulative effect of linear, quadratic *el-ph* interaction strengths and RSOI on ground state energy. We have observed that ground state energy decreases with $g_{1}$ due to polaronic effect. Additionally, it is discovered that the ground state energy is lower for negative values of $g_{2}$ than for positive ones. For positive (negative) quadratic *el-ph* interaction has been found to diminish (increase) the effective coupling between the electrons and phonons and to harden (soften) the effective phonon frequency. It demonstrates that in systems with negative $g_2$, the polaronic effects are stronger.

**Fig. 3:**
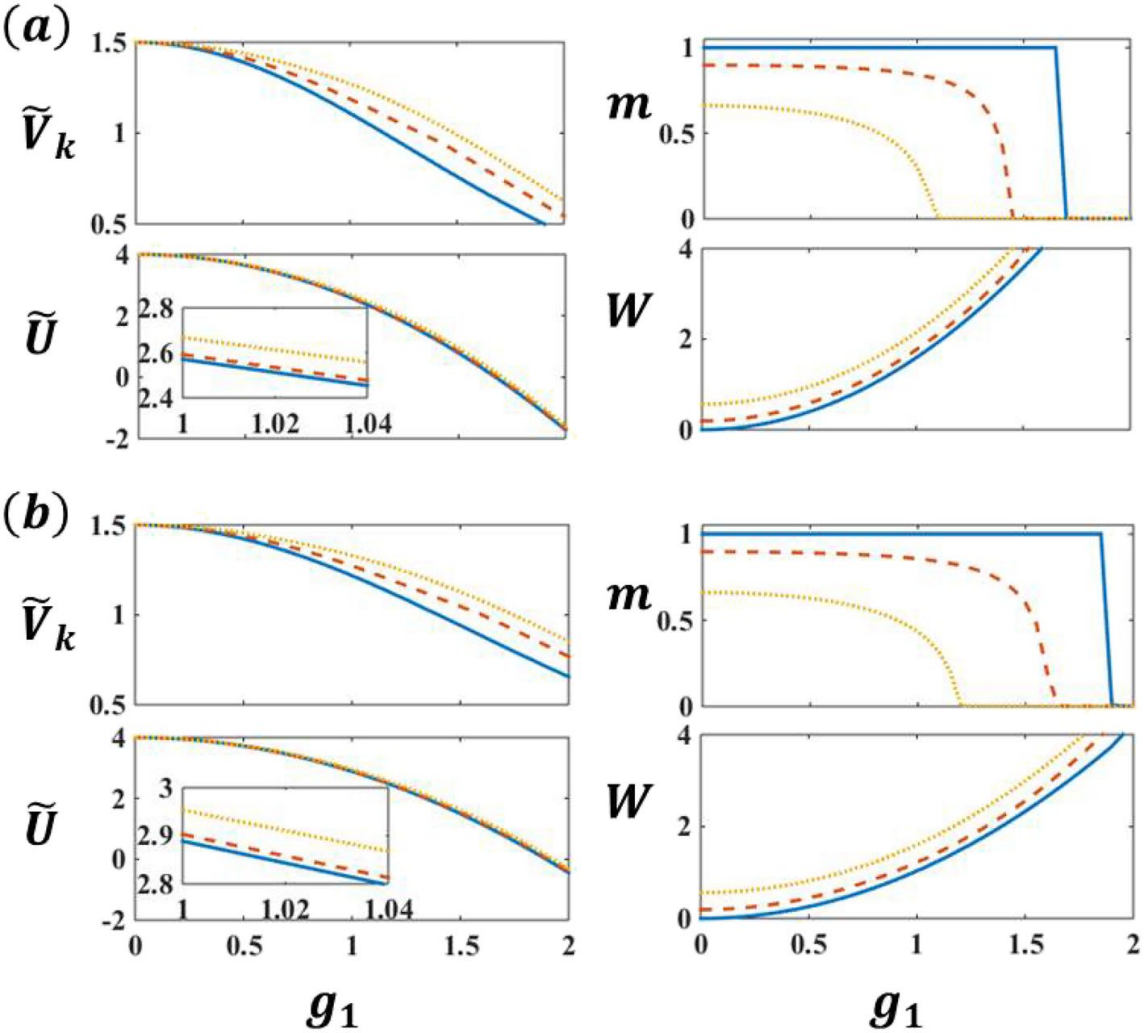
Variation of renormalized tunnelling strength, Coulomb interaction strength, local magnetic moment, and binding energy of the bound state as a function of $g_{1}$ for three different values of $K_{r}L=0$ (solid), $0.7\pi$ (dashed), $\pi$ (dot-dashed). $(a)\, g_{2}=0.1 \, (b)\, g_{2}=0.2$. It is found that as $g_1$ increases $\tilde{V_k}$, $\tilde{U}$, *m* decreases and *W* increases.

**Fig. 4:**
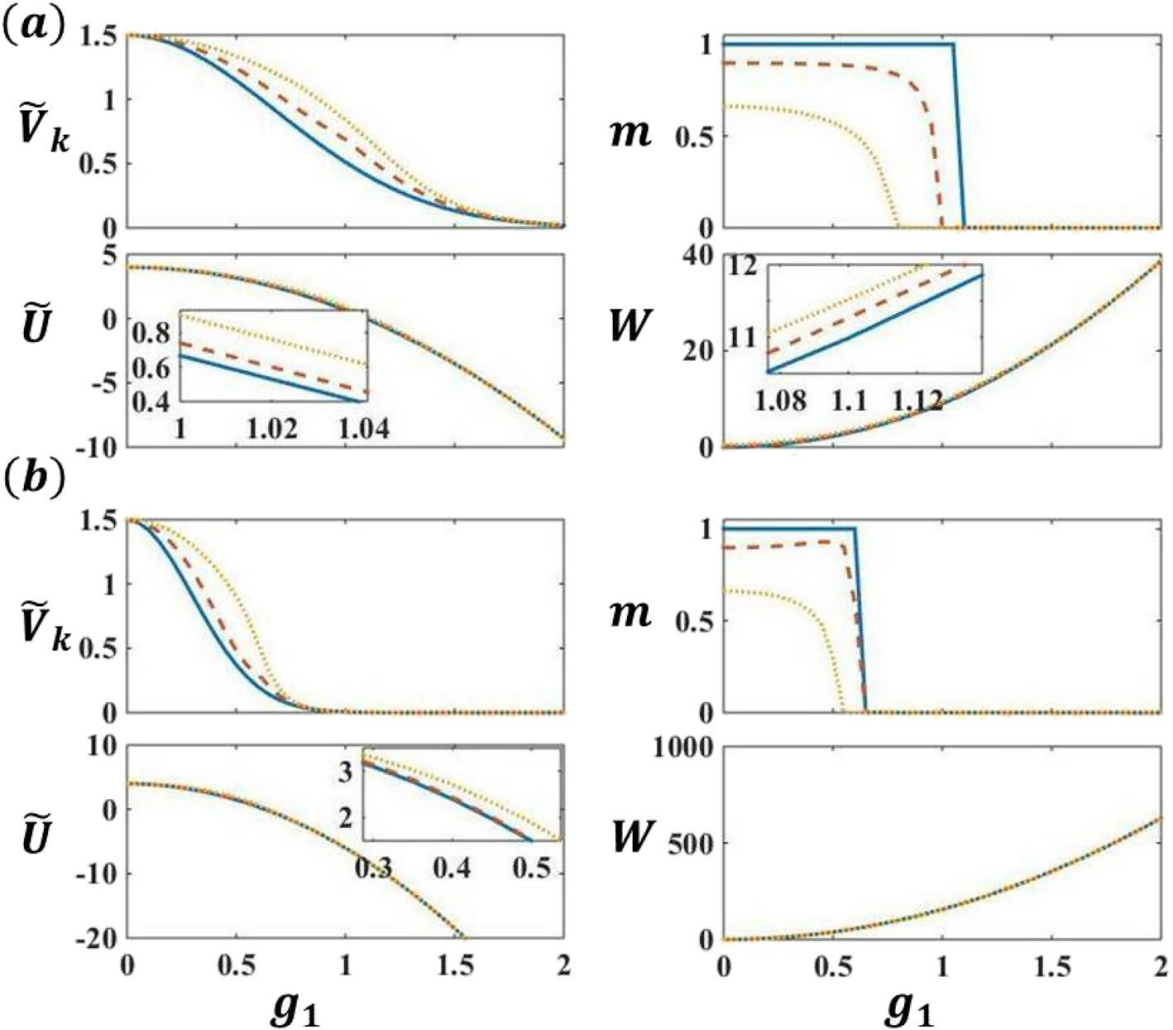
Variation of renormalized tunnelling strength, Coulomb interaction strength, local magnetic moment, and binding energy of the bound state as a function of $g_{1}$ for three different values of $K_{r}L=0$ (solid), $0.7\pi$ (dashed), $\pi$ (dot-dashed). $(a)\, g_{2}=-0.1 \, (b)\, g_{2}=-0.2$. It is found that as $g_1$ increases $\tilde{V_k}$, $\tilde{U}$, *m* decreases and *W* increases.

Results for the renormalized hopping and Coulomb interaction parameters, local magnetic moment, and binding energy of the bound state formed at the impurity site as a function of $g_{1}$ for three distinct values of the RSOI with positive and negative values of the quadratic *el-ph* interaction $g_{2}$ are shown in Figs. 3 and 4 respectively. We observed that the local magnetic moment value decreases, and the binding energy increases as the linear *el-ph* interaction strength increases due to the polaronic effect. The tunneling strength enhances the hybridization between conduction electrons and localized impurity electron. As a result, binding energy increases with increasing the tunneling strength. On the other hand, Coulomb interaction reduces the double occupancy on the impurity. As a result, the local magnetic moment increases with increasing *U*. Interestingly we have noticed that the local magnetic moment value decreases, and the binding energy increases as the RSOI increases. As we have already mentioned, the RSOI enhances the mixing of spin-up and spin-down states between conduction and impurity electrons at the QD. This gives more scope to the hybridization, reducing the magnetic moment and increasing the binding energy. It shows that local magnetic moment and the binding energy of the impurity bound state are competing factors. Also, the critical value of $g_{1}$ (at which $m=0$) is decreasing with an increase in RSOI. It is easy to observe that the increases in RSOI cause $\tilde{V}_{k}$ to rise more similarly to increases in $\tilde{U}$. As we have already explained positive (negative) $g_{2}$ enhances the polaronic effects by hardening (softening) the phonon mode. Our results show that the RSOI enhances the binding energy of the impurity-bound state by reducing the local magnetic moment.

**Fig. 5:**
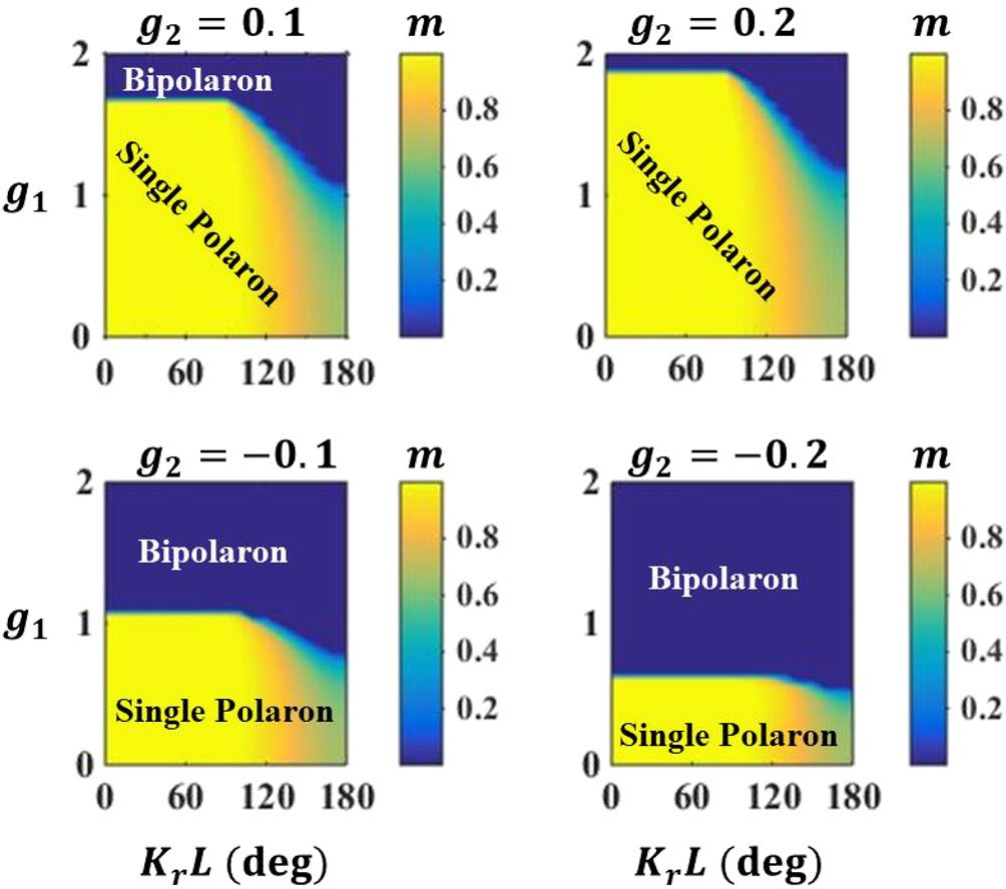
Colormap phase diagram of local magnetic moment as a function of $g_1$ and $K_{r}L$ to indicate the possible regions for the formation of bipolaron $(W\ne 0)$ and polaron $(W=0)$ states for both positive and negative values of $g_{2}$. We found that the increasing linear *el-ph* interaction $g_1$ and RSOI strength $K_{r}L$ enhance the formation of the bipolaronic phase at the impurity site by reducing the *m*. Additionally, negative (positive) quadratic *el-ph* favors the formation of the bipolaronic (single polaronic) phase at the impurity.

**Fig. 6:**
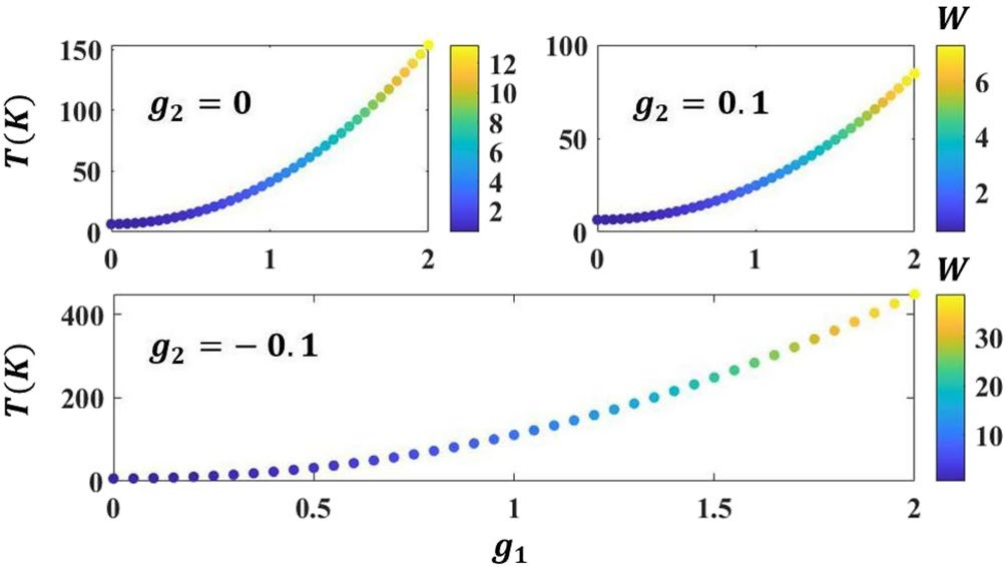
Variation of *W* as a function of $g_1$ and *T* for $K_{r}L=\pi$ and three different values of $g_{2}$. It is discovered that *W* and *T*(*K*) grow as $g_1$ increases.

In Fig. 5, we present the phase diagram result for the magnetic moment as a function of $g_1$ and RSOI strength for $g_{2}=\pm 0.1,\,\pm 0.2$ to investigate the cumulative effect of $g_1$ and RSOI on the impurity bound state. It shows that quadratic *el-ph* interaction with positive (negative) coupling constant enhances the formation of polaron (bipolaron) instead of bipolaron (polaron) at the impurity site. In the region, where $m=1$ ($m=0$) which can be described as a single polaron (bipolaron) state. Stated differently, “impurity in a Kondo type singlet (magnetic doublet) state with *el-ph* interaction” describes a magnetic impurity whose spin is paired with the spins of the conduction electrons in the host BCS superconductor, producing a net magnetic moment of zero (non-zero), can refer to the formation of bipolaron (single polaron). It is interesting to note that the RSOI strength allows the formation of an impurity bound state by enhancing the polaronic effects. By adjusting the Rashba interaction’s strength, one can regulate the impurity bound state’s binding energy and magnetic moment magnitude. We have finally shown the binding energy as a function of $g_{1}$ and the critical temperature at which the bound state breaks for $K_{r}L=\pi$ in Fig. 6. It indicates that more thermal energy is required to break the impurity-bound state as $g_{1}$ rises. Our research will contribute to the understanding of current attempts to realise topological superconducting phases with Majorana end states by using magnetic atom chains deposited on the surface of a superconductor with a strong SOC, like Pb^18^.

**Fig. 7:**
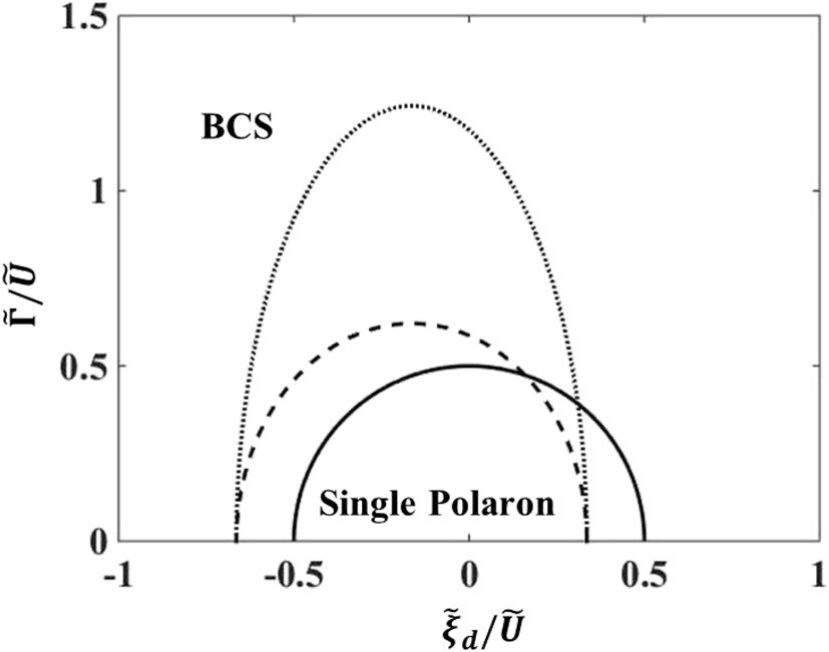
Phase diagram of a impurity as a function of renormalized Coulomb interaction *U*, energy level ${\tilde{\xi }}_d$ and hybridization ${\tilde{\Gamma }}$ in the subgap limit or large gap limit. Area under the transition line represents the single polaron phase. Here solid line represents $g_{1}=g_{2}=K_{r}L=0$, dashed line represents $g_{1}=0.6,\,\,g_{2}=0.1,\,\,K_{r}L=0$, and dotted line corresponds to $g_{1}=0.6,\,\,g_{2}=0.1,\,\,K_{r}L=\pi /4$.

To investigate the phase transition at impurity in the subgap limit, we have considered $\Delta _s \rightarrow \infty$, as a result the quasiparticles in the superconductor are inaccessible and the coupling between them and the impurity vanishes, and the impurity will be proximitized by the Cooper pairs at the Fermi level. The quantum impurity’s state in the large-gap limit is thus the consequence of a competition between the Coulomb interaction and the local pairing strength $\tilde{\Gamma }$ induced by the proximity effect. From Fig. 7, we can observe that the increase in the strength of the RSOI enhances the formation of a single polaron at the impurity.

## Summary and conclusion

In this work, We have studied the effect of spin-orbit coupling on the impurity-induced bound states (YSR states) in BCS superconductors along with both linear and quadratic *el-ph* interactions using the spin-degenerate Anderson-Holstein model with the BCS term. Our thorough approach involved the use of the cluster variation method to calculate the system’s ground state energy. We further calculated the binding energy of the bound state and the local magnetic moment formed at the impurity site. The effective phonon mode frequency is increased (decreased) by the quadratic *el-ph* interaction with a positive (negative) coupling constant. We found that the effective linear *el-ph* interaction strength is reduced (increased) by a quadratic *el-ph* interaction with a positive (negative) coupling constant. Also, we have demonstrated a rise in the quadratic *el-ph* interaction with positive and negative, which promotes the polaron and bipolaron formation at the impurity site. Additionally, we have seen that the impurity transitions from a BCS-like state to a single polaron state in the subgap limit when the RSOI increases. Our findings demonstrate the ability to control the binding energy and magnetic moment magnitude of the impurity bound state by varying the strength of the RSOI. Our system, a single magnetic impurity (magnetic QD with *el-ph* and RSOI) embedded within a superconductor, effectively mimics the behaviour of the SC-QD-SC device. So our theoretical results can be easily verified in the laboratory and mimics an SC-QD-SC device with Nitro-substituted Oligo (phenylene ethynylene) (OPE-NO2) molecules^51^, *FeSe*^45^, $MgB_{2}$^52^ as a QD. Also, our theoretical findings may be experimentally confirmed for a magnetic QD deposited on a BCS superconductor by measuring the density of a QD using spectroscopic methods such as tunnelling spectroscopy, Andreev reflection spectroscopy, etc.

## Data Availability

No datasets were generated or analysed during the current study.
